# *ZjHXK5* and *ZjHXK6* negatively regulate the sugar metabolism of *Ziziphus jujuba* Mill.

**DOI:** 10.3389/fpls.2024.1335120

**Published:** 2024-02-12

**Authors:** Panpan Tong, Guanglian Liao, Dengyang Lu, Xiaofeng Zhou, Wang Zhang, Qiang Xu, Cuiyun Wu, Jiangbo Wang

**Affiliations:** ^1^ College of Life Science and Technology, Tarim University, Alar, Xinjiang, China; ^2^ National-Local Joint Engineering Laboratory of High Efficiency and Superior Quality Cultivation and Fruit Deep Processing Technology on Characteristic Fruit Trees, Alar, Xinjiang, China; ^3^ National Key Laboratory for Germplasm Innovation & Utilization of Horticultural Crops, Huazhong Agricultural University, Wuhan, Hubei, China; ^4^ College of Horticulture and Forestry, Tarim University, Alar, Xinjiang, China

**Keywords:** bioinformatics analysis, hexokinase, jujube, methyl jasmonate, sugar metabolism

## Abstract

Hexokinase (HXK) plays a crucial role in plants, catalyzing the phosphorylation of hexose substances, which is one of the key steps in sugar metabolism and energy production. While *HXK* genes have been well-studied in model plants, the evolutionary and functional characteristics of HXK gene family in jujube is unknow. In this study, the *HXK* gene family members were identified by bioinformatics methods, the key members regulating glucose metabolism were identified by transcriptome data, and finally the function of the key genes was verified by instantaneous and stable genetic transformation. Our results showed that seven *HXK* genes were identified in the jujube genome, all of which were predict located in the chloroplast and contain Hexokinase-1 (PF00349) and Hexokinase-2 (PF03727) conserved domains. Most of HXK proteins were transmembrane protein with stable, lipid-soluble, hydrophilic. The secondary structure of ZjHXK proteins main α-helix, and contains two distinct tertiary structure. All *ZjHXK* genes contain nine exons and eight introns. Predictions of cis-regulatory elements indicate that the promoter region of ZjHXK contains a large number of MeJA responsive elements. Finally, combined with the analysis of the relationship between the expression and glucose metabolism, found that *ZjHXK5* and *ZjHXK6* may the key genes regulating sugar metabolism. Transient overexpression of *ZjHXK5* and *ZjHXK6* on jujube, or allogeneic overexpression of *ZjHXK5* and *ZjHXK6* on tomato would significantly reduce the content of total sugar and various sugar components. Transient silencing of *ZjHXK5* and *ZjHXK6* genes results in a significant increase in sucrose and total sugar content. Interestingly, the expression of *ZjHXK5* and *ZjHXK6* were also affected by methyl jasmonate.

## Introduction

*Ziziphus jujuba* Mill. is origin from China and widely distributed in temperate and subtropical regions of the Northern Hemisphere (Huang, 2020). Jujube fruits are rich in nutrition, especially the ascorbic acid, amino acid and sugar (Liu et al., 2020; Liao et al., 2023). The sugar content in ripe fresh jujube fruits is up to 25%~40%, which is a traditional fruit with the same origin as medicine and food (Li et al., 2007; Chen and Tsim, 2020). The rich germplasm resources and the variation of sugar content make jujube an important species for studying sugar metabolism.

In higher plants, sugar can not only be used as an energy source and structural component, but also participate in the growth and development process as a transmission signal (Jang and Sheen, 1994). Sugar metabolism is regulated by several enzymes, among which hexokinase (HXK) is closely related to glucose metabolism and as a hexose sensor, *HXK* can senses the levels of hexoses and phosphorylation states. Then through signal transduction pathways, *HXK* convey information to the cell nucleus, this process playing a crucial role in sugar sensing and signaling (Moore et al., 2003; Rolland et al., 2006). Importantly, *HXK* participates in sucrose-induced signal transduction and regulates the expression of genes related to sucrose metabolism and starch synthesis (Xiao et al., 2000; Polit and Ciereszko, 2009). For example, reduced *StHXK1* activity leads to accumulation of glucose and starch, and decreased sucrose levels (Veramendi et al., 2002). Hexokinase-like (HKL) is an isoenzyme of *HXK*, and although the function of HLK is unknown, defective phosphorylation of *AtHKL1* can negatively regulate plant growth (Karve et al., 2008). Recent studies also indicate that *HXK* effect root development (Granot et al., 2014), leaf senescence (Swartzberg et al., 2011), regulates photosynthesis (Roth et al., 2019), pollen germination (Karni and Aloni, 2002), and sugar content during fruit development (Ji et al., 2023).

Currently, *HXK* genes have been isolated from many plants, including pear (Zhao et al., 2019), apple (Zhu et al., 2021), and peach (Xu et al., 2021), existing in the form of gene families in plants (Karve et al., 2010). Six and ten *HXK* gene family members have been identified in *Arabidopsis thaliana* (Karve et al., 2008) and rice (Cho et al., 2006) respectively. Different *HXK* members showed tissue-specific expression. For example, *AtHXK1* was expressed in all organs, while *AtHXK3* showed higher expression in roots and siliques (Karve et al., 2008).

In addition, the sugar content and *HXK* expression were induced by abiotic stress, such as temperature and methyl jasmonate (Sami et al., 2016; Saddhe et al., 2021). In model plants, the structure and function of HXK have been well understood. However, the identification and there few reports on the functional analysis of HXK gene in jujube. Therefore, the aim of this study was to identify the members of the *ZjHXK* gene family at the whole genome level, and to clarify the function of key genes regulating glucose metabolism and their response to abiotic stress.

## Materials and methods

### Materials and treatments

Different developmental stages of cv ‘Fucuimi’ (higher sugar content) and cv ‘Jing 39’ (lower sugar content) fruit were harvested, including the young fruit stage (YF), early white maturation stage (EF), white maturation stage (WM), half-red fruit stage (HR), and full-red fruit stage (FR). Nine cv ‘Fucuimi’ and nine cv ‘Jing 39’ were cultivated at the Tarim University Jujube Germplasm Resource Repository (40°54′N, 81°30′E), with uniform cultivation conditions. Three plants were a biological replicate, and each plant collected 15 fruits from four directions (east, south, west, and north). The collected samples were treated with liquid nitrogen at once, and stored at -80°C until use. The Beijing Novogene Biotech Co., Ltd. performed sequencing with HiSeq2500, the winter jujube genome (PRJNA251714) (Liu et al., 2014) was used as reference genome. Transcriptome data of different winter jujube tissues were downloaded from the NCBI database (PRJNA260241).

‘Jing39’ jujube callus tissues were used as the experimental material for different concentration MeJA treatment, including control (0 mmol/L), T1 (0.05 mmol/L), T2 (0.10 mmol/L), T3 (0.20 mmol/L) and T4 (0.30 mmol/L). Each treatment was repeated three times, and the callus was treated in 25°C darkness for 10 d, and then frozen in liquid nitrogen.

### Identification of ZjHXK

Firstly, the jujube genome (PRJNA251714) was downloaded from National Center for Biotechnology Information database, and then the protein sequence of AtHXK in *Arabidopsis* was used as a probe to blast in jujube proteins to obtain ZjHXK candidate sequence (Liu et al., 2014). The ZjHXK candidate sequences that contain the Hexokinase-1 (PF00349) and Hexokinase-2 (PF03727) domain were considered the *ZjHXK* gene family members by the Pfam database (http://pfam.xfam.org/).

### Bioinformatics analysis

TMHMM Serverv. 2. R2.0 (http://www.cbs.dtu.dk/services/TMHMM) and ProtParam (https://web.expasy.org/protparam/) predicted *ZjHXK* physicochemical properties. WoLF PSORT (https://wolfpsort.hgc.jp/) predicted the subcellular localization of *ZjHXK*. NPSA (https://npsa-prabi.ibcp.fr/cgi-bin/npsa_automat.pl?page=npsa_sopma.html) and Swiss model (https://swissmodel.expasy.org/) predicted the secondary structure and tertiary structure of ZjHXK, respectively. Signalp 6.0 (https://services.healthtech.dtu.dk/services/SignalP-6.0/) and Netphos 3.1 (https://services.healthtech.dtu.dk/services/NetPhos-3.1/) predicted signal peptide, phosphorylation and glycosylation sites, respectively. The TBtools-II (Chen et al., 2023) was used to extract genome, promoter and CDS sequences, and the gene structure display by GSDS (http://gsds.gao-lab.org/index.php) (Hu et al., 2015). PlantCARE (http://bioinformatics.psb.ugent.Be/webtools/PlantCARE/HTML/) was used for predicted the cis regulatory elements. MEME (https://meme-suite.org/meme/tools/meme) (Bailey et al., 2006) predicted conservative motifs with 10 ordinals and 5-50 aa length. The ClustalW method in MEGA11.0 software was used to blast, then constructed phylogenetic tree with the Neighbor-Joining method, the bootstrap validation value was set to 1,000. The phylogenetic tree was beautified by Evolview (https://evolgenius.info//evolview-v2). TBtools-II (Chen et al., 2023) was used to conduct multicollinearity analysis among gene family members.

### Cloning and vector construction of *ZjHXK5* and *ZjHXK6*

Specific primers for the full-length coding sequences (CDS) of *ZjHXK5* (GenBank accession number: XM_048465612.1) and *ZjHXK6* (GenBank accession number: XM_016018332.3) genes were designed using Primer5.0 software. The primer sequences were as follows: For *ZjHXK5* CDS, the forward primer was *ZjHXK5*-CDS-F: ATGGGGAAATTGGCGGTGGGTG, and the reverse primer was *ZjHXK5*-CDS-R: TTAAGACTCTTCTACCTCTAAG. For *ZjHXK6* CDS, the forward primer was *ZjHXK6*-CDS-F: ATGGGGAGGGTGGTGGTGGGAG, and the reverse primer was *ZjHXK6*-CDS-R: CTATCCACACTGTTGGATGTTG. Total RNA from mature ‘Jing39’ fruit at the ripe stage was extracted using the TIANGEN RNAprep Pure Polysaccharide and Polyphenolics Plant Total RNA Extraction Kit (Tiangen Biochemical Technology Co., LTD., Beijing, China). Subsequently, cDNA was synthesized by the HiScript II Q RT SuperMix for qPCR (+gDNA wiper) kit (Novozymes Biotechnology Co., Ltd., Nanjing, China). Gene cloning and LR reaction refer to the previous method (Wasaya et al., 2023) to construct CDS sequences of *ZjHXK5* and *ZjHXK6* into pK7WG2D vector. Tsingke Biotech Co., Ltd. (Wuhan, China) performed the sequencing. The sequencing confirmed cloned plasmids were extracted using the SIMGEN Rapid Plasmid Mini Kit (Xinjing biological reagent development Co., LTD., Wuhan, China). VIGS-mediated silencing of *ZjHXK5* and *ZjHXK6* gene expression in jujube fruits according to the previous methods (Tian et al., 2014).

### Transient and stable transformation

The plasmids confirmed by sequencing were named pK7WG2D-*ZjHXK5-GFP*, pK7WG2D-*ZjHXK6-GFP* and *pTRV2-ZjHXK5, pTRV2-ZjHXK6*, and transformed into *Agrobacterium* strains GV3101 and EHA105. *Agrobacterium* strains GV3101 carrying pK7WG2D-*ZjHXK5-GFP* and pK7WG2D-*ZjHXK6-GFP* were separately used for injection into one-month-old tobacco leaves (Sparkes et al., 2006) and cv ‘Jing39’ ripening jujube fruits with similar size. After injection, dark treat for 1 d, followed by normal light treatment for 2 d. *Agrobacterium* strains GV3101 carrying *pTRV2-ZjHXK5* and *pTRV2-ZjHXK6*, were separately used for injection into cv ‘Dongzao’ ripening jujube fruits with similar size. After injection, dark treat for 1 d, followed by normal light treatment for 4 d (Tian et al., 2014). *Agrobacterium* EHA105 carrying *pK7WG2D-ZjHXK5-GFP* and *pK7WG2D-ZjHXK6-GFP* were used for infection of Micro-Tom tomato (Sun et al., 2015) and ‘Jing39’ jujube callus, respectively. Fruits and callus were collected from T2 generation positive lines.

### Determination of the sugar component content

Sugar component extraction was performed following the method described by previous methods (Bartolozzi et al., 1997). Previous studies have indicated that the major sugar components in jujube fruits include fructose, glucose, and sucrose (Hubbard et al., 1991; Li et al., 2007; Rashwan et al., 2020), accounting for over 95% of the total sugar content. Therefore, in this study, the total content of these three sugar components was considered as the total sugar content.

### Expression analysis by qRT-PCR

Following previous method, the *ZjUBQ* was used as the internal reference gene for qRT-PCR analysis (Zhang et al., 2015). A reaction mixture composed of 10 μL as follows: 0.5 μL of cDNA template, 0.2 μL for each forward and reverse primer, 5 μL of PowerUp TM SYBR TM Green Master Mix, and 4.1 μL of ddH_2_O. The 2^^-ΔΔCT^ method was used for calculate the relative gene expression levels (Livak and Schmittgen, 2001).

### Data analysis

The data was statistically analyzed by GraphPad Prism (Mitteer and Greer, 2022). Duncan’s method was used to detect differences at *P* ≤ 0.05. The heat map of expression was drawn with TBtools-II (Chen et al., 2023).

## Results

### Identification and characterization of HXK gene family members in jujube

A total of seven ZjHXK candidate sequences were confirmed, all of them contain conserved domains PF00349.24 and PF03727.19 (**Supplementary Figure 1**). It’s worth noting that these candidate sequences were distributed non-uniformly across chromosomes 1, 6, 8, 12 and an unidentified chromosome, and they were named as ZjHXK1to ZjHXK7 based on their chromosomal locations. The physicochemical properties of ZjHXK (**Table 1**) showed that coding sequence (CDS) lengths ranged from 1473 to 1521bp. The molecular weights of ZjHXK proteins varied from 52.98 to 55.05 kDa, and the isoelectric points (pI) ranged from 5.52 to 7.65. Notably, ZjHXK2 protein was predicted located extracellularly and not a transmembrane protein. All but ZjHXK6 and ZjHXK7 were stable proteins. All ZjHXK proteins were lipophilic protein. except ZjHXK2, other ZjHXK was hydrophilic protein. Subcellular prediction analysis revealed that all ZjHXK were localized within the chloroplast.

**Table 1:** Physicochemical properties of HXK gene family proteins.

Gene name	Gene ID	CDS (bp)	Protein (aa)	Position	Formula	MW (kDa)	TMs	PI	Instability Index	Aliphatic Index	Grand Average of Hydropathicity	Subcellular predictive localization
ZjHXK1	LOC107435312	1494	498	Chr1:46678375-46683614+	C4488H7481N1497O1904S282	53.75	1	5.84	32.08	96.18	-0.001	Chloroplast
ZjHXK2	LOC107435837	1479	493	Chr6:1387393-1390742-	C4428H7376N1482O1868S300	52.98	0	5.52	37.08	93.98	0.034	Chloroplast
ZjHXK3	LOC107425017	1473	491	Chr8:12801933-12805086-	C4415H7356N1476O1863S263	54.10	1	6.11	35.38	94.09	-0.125	Chloroplast
ZjHXK4	LOC107435723	1497	499	Chr12:14728722-14735939-	C4463H7428N1500O1887S287	54.23	1	5.96	33.64	93.83	-0.069	Chloroplast
ZjHXK5	LOC125419476	1494	498	Chr12:14928100-14935202-	C4449H7403N1497O1882S288	54.07	1	5.91	33.77	93.63	-0.068	Chloroplast
ZjHXK6	LOC107410849	1521	507	ChrUn1:210699-216640-	C4521H7520N1524O1934S246	55.05	1	7.65	47.98	93.98	-0.016	Chloroplast
ZjHXK7	LOC125419907	1521	507	ChrUn2:85255-91192+	C4521H7520N1524O1934S246	55.05	1	7.65	47.98	93.98	-0.016	Chloroplast

Instability index more than 40 means unstable; aliphatic index less than 100 means lipid soluble protein; value of grand average of hydrophobicity being positive means hydrophobicity, while negative means hydrophilicity.

### Protein signaling peptides, glycosylation and phosphorylation sites analysis

The signal peptide analysis of ZjHXK proteins revealed that all ZjHXK protein can consider as non-secretory proteins (**Supplementary Figure 2**). Phosphorylation site analysis found that ZjHXK proteins had many multiple potential phosphorylation sites (**Supplementary Figure 3**). such as ZjHXK6 and ZjHXK7 exhibited the highest number of phosphorylation sites, including 29 Serine (Ser), 15 Threonine (Thr), and 3 Tyrosine (Tyr) phosphorylation sites. Glycosylation sites analysis of ZjHXK proteins (**Supplementary Figure 4**) indicated that ZjHXK1 had a single typical N-glycosylation site located at amino acid position 229. In contrast, ZjHXK2 lacks N-glycosylation sites.

### Prediction of secondary and tertiary structure of proteins

**Figure 1:**
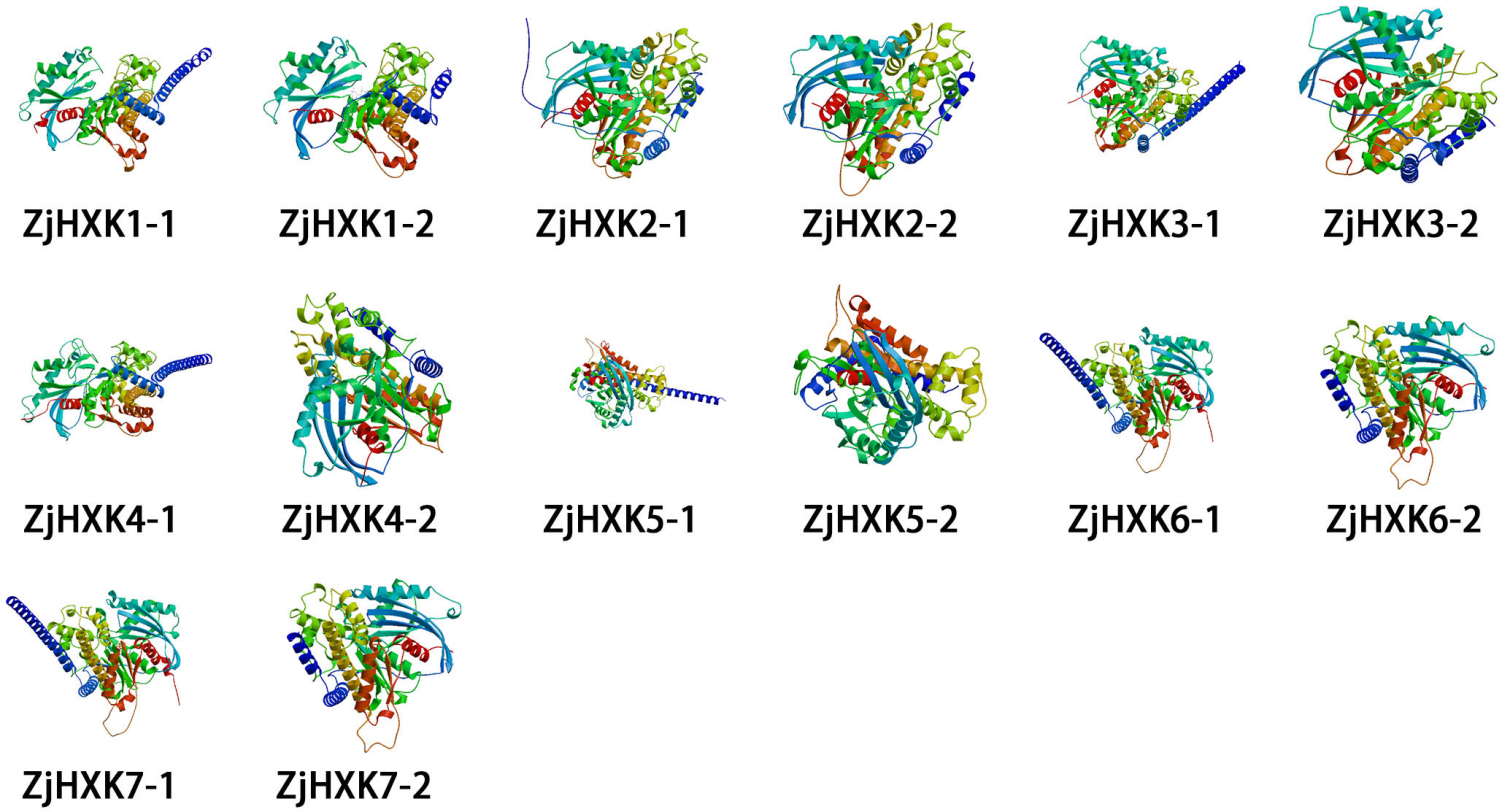
Tertiary structure analysis. There may be multiple tertiary structure models for the same protein sequence.

The secondary structure predictions of ZjHXK proteins indicate that the ZjHXK protein were composed of α-helices, random coils, extended strands, and β-turns. Among them, the α-helices was the main component, ranging from 40.97% to 47.25% (**Supplementary Figure 5**). Each ZjHXK protein contains two distinct tertiary structures, with significant differences between them (**Figure 1**).

### Phylogenetic analysis

**Figure 2:**
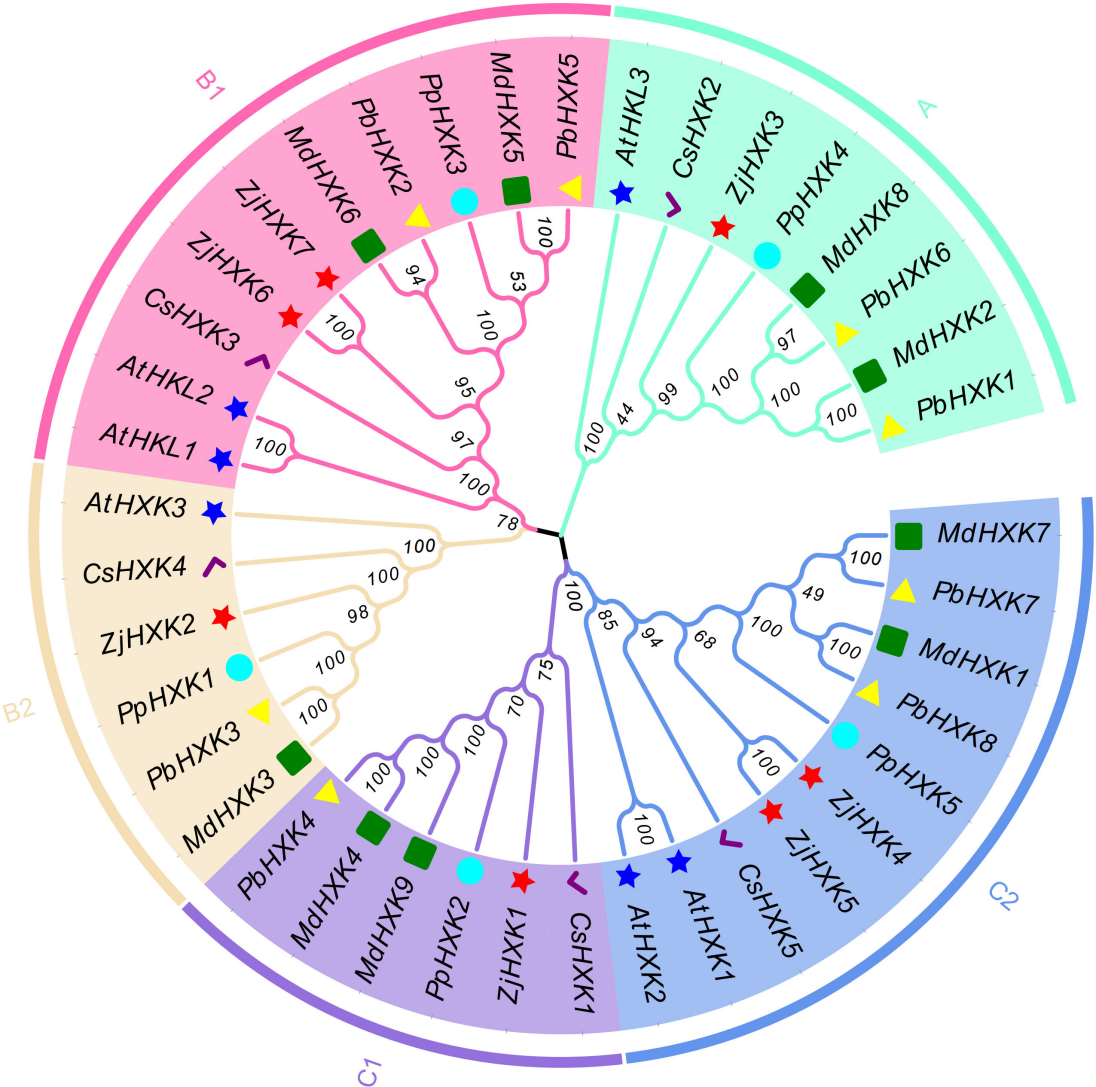
Phylogenetic analysis of HXK proteins from Jujube and other plants. *At*, *Arabidopsis thaliana*; *Md*, *Malus domestica*; *Pp*, *Prunus persica*; *Pb*, *Pyrus bretschneideri*; *Cs*, *Citrus sinensis*. The red star, the yellow triangle, the blue star, the green rect, the cyan circle and the purple tick were represented respectively *Ziziphus jujuba*, *Pyrus bretschneideri*, *Arabidopsis thaliana*, *Malus domestica*, *Prunus persica* and *Citrus sinensis*. A, B and C represent the three groups, respectively.

The protein sequences of *Arabidopsis* HXK (AtHXK), apple HXK (MdHXK), peach HXK (PpHXK), pear HXK (PbHXK), and sweet orange HXK (CsHXK) were used to construct a neighbor-joining phylogenetic tree (**Figure 2**). These genes were classified into groups A, B and C, the group B and groups C further divided into two subgroups. Among them, ZjHXK members were distributed in all five subgroups, with ZjHXK3 in group A, ZjHXK6 and ZjHXK7 in subgroup B1, ZjHXK2 in subgroup B2, ZjHXK1 in subgroup C1, ZjHXK4 and ZjHXK5 in subgroup C2. Group A genes were mostly alkaline proteins, subgroup B1 protein were non-transmembrane proteins, subgroup B2 proteins have the highest proportion of extended chains, subgroup C1 contains more cis-acting elements related to stress response, and C2 subgroup proteins have the highest proportion of α-helices.

### Gene structure and conserved motifs of ZjHXK genes

**Figure 3:**
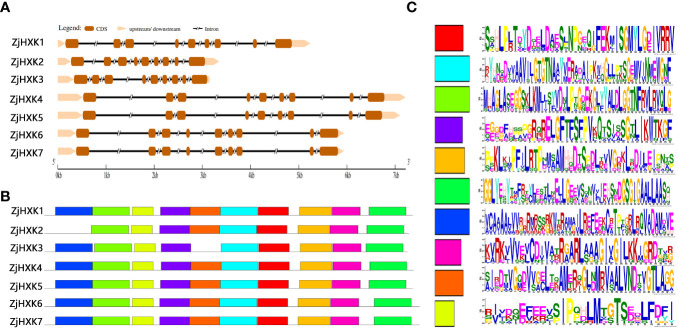
The gene structure and conserved motifs of *ZjHXK* genes in jujube. The gene structure of *ZjHXK* genes **(A)**, in which brown lines represent exons and black lines represent introns. The conserved motifs of ZjHXK proteins **(B)**, in which the conserved motifs are indicated by colored boxes. Sequences of the 10 conserved motifs in the ZjHXK proteins **(C)**.

The gene structure analysis (**Figure 3A**) showed that each of the *ZjHXK* genes contained nine exons separated by eight introns. Among these, *ZjHXK3* exhibits the shortest gene length, while longest for *ZjHXK4*. The MEME of ZjHXK proteins found that 10 putative conserved motifs were identified in all ZjHXK proteins except ZjHXK2 and ZjHXK3. In addition, the ZjHXK2 gene lacked motif 7, and the ZjHXK3 gene lacked motif 9 (**Figure 3B**). The lengths of the conserved motifs ranged from 28 to 50 aa (**Figure 3C**).

### Promoter cis-element analysis

**Figure 4:**
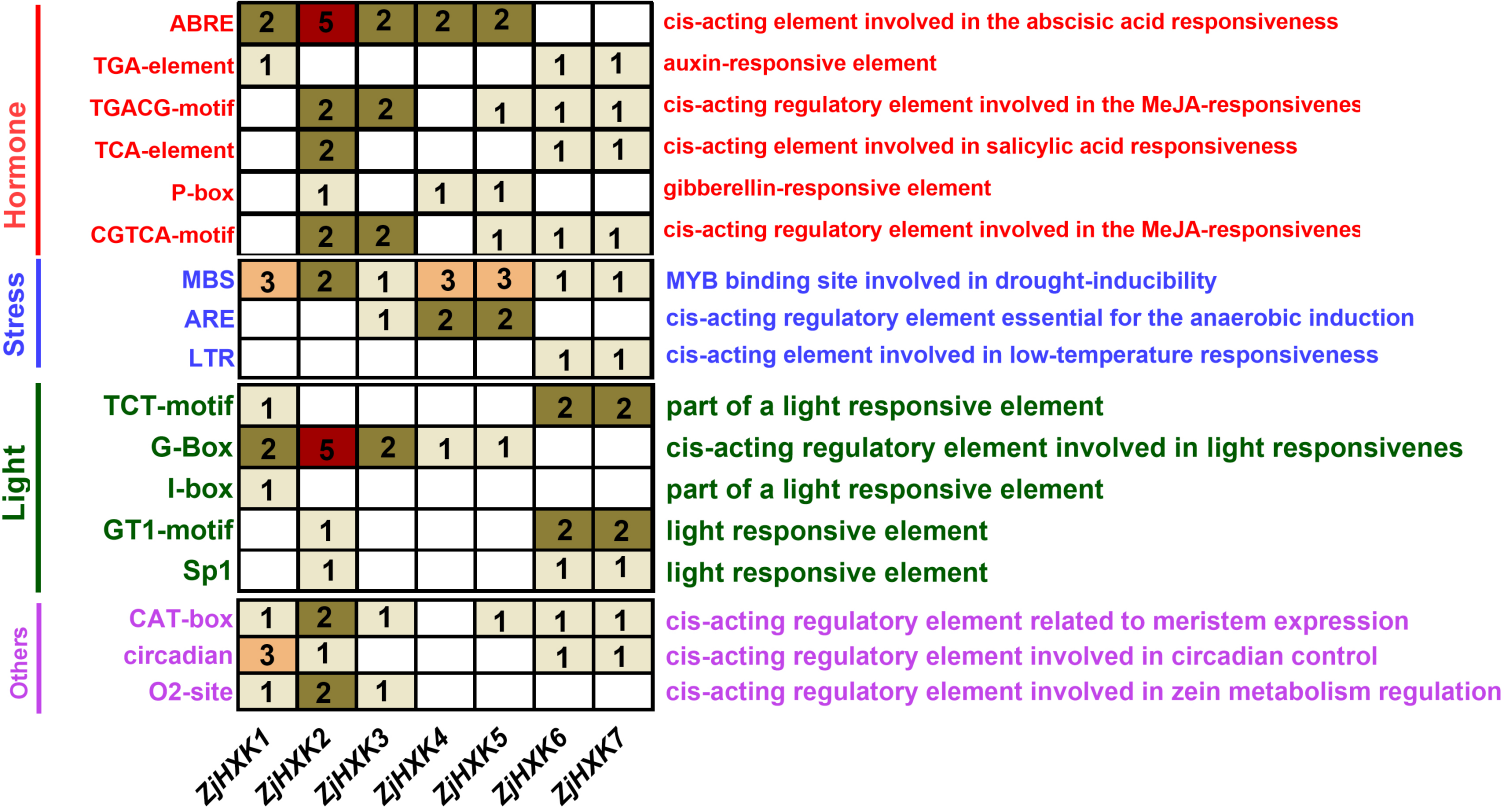
Promoter cis-element analysis of *HXK* gene family. The figure does not show all cis-acting elements, but only the cis-acting elements that are prevalent on *ZjHXK*. The cis-acting elements related to hormones were shown in red, those related to stress were shown in blue, and those related to light were shown in green. The others are shown in purple. The number in the box represents the number of corresponding cis-acting elements in the corresponding promoter sequence.

Promoter cis-acting regulatory elements results showed the there were many cis-acting regulatory elements involved in hormones, stress, and light in the promoter regions (**Figure 4**). Among these elements, there were relatively more cis-acting regulatory elements associated with hormone response in *ZjHXK* promoter regions, while the number of cis-acting regulatory elements related to stress response was relatively lower. It is worth noting that the MeJA-responsiveness cis-acting elements, linked to hormone response, were the most abundant. In addition, *ZjHXK2* displayed the highest number of ABRE and G-Box cis-acting elements, whereas *ZjHXK4* and *ZjHXK5* had only one cis-acting element related to light response but the highest number of cis-acting elements associated with stress response.

### Intragenomic and intergenomic collinearity analysis

**Figure 5:**
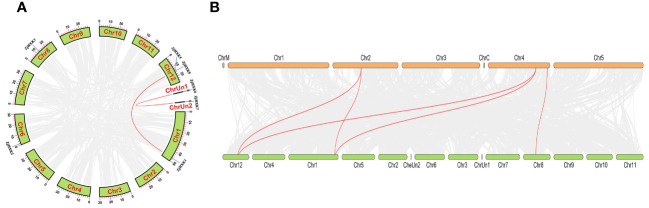
Collinearity analysis of the HXK gene families in *Ziziphus jujuba* **(A)**, between *Ziziphus jujuba* and *Arabidopsis* **(B)**. The red lines connect two genes which exist multicollinearity. In a, the squares around the circles represent 12 chromosomes of *Ziziphus jujuba*. Among them, Ziziphus jujuba has two sequence that has not been assembled into chromosomes. In b, the green boxes represent chromosomes of Ziziphus jujuba. The orange boxes represent the chromosomes of *Arabidopsis*.

Through intragenomic collinearity analysis, two pairs of collinear gene pairs were identified, namely, ZjHXK1 and ZjHXK5, and ZjHXK6 and ZjHXK7 (**Figure 5A**). Through intergenomic collinearity analysis, genetic differences and gene duplications of HXK genes between jujube and *Arabidopsis* were explored. Collinearity was found between four ZjHXK gene family members and three *Arabidopsis* HXK genes (**Figure 5B**). Specifically, ZjHXK4 (chr 12), ZjHXK5 (chr 12) and ZjHXK1 (chr 1), exhibit collinearity with AtHXK2 (chr 2) and AtHXK1 (chr 4). ZjHXK3 (chr 8) shared collinearity with AtHKL3 (chr 4). Additionally, during the evolutionary process in different species, duplicated genes may have undergone changes or been lost.

### Sugar composition and *ZjHXK* gene expression patterns in different developmental stages of fruit

**Figure 6:**
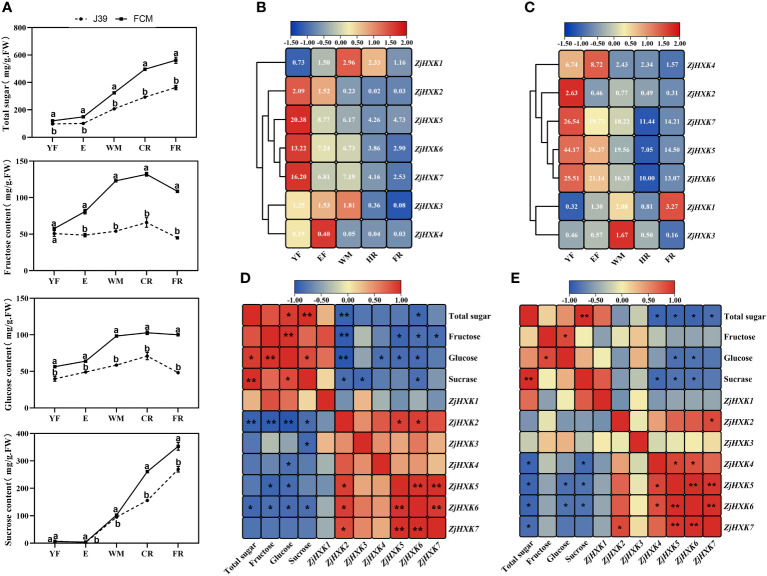
Sugar content of ‘Fucuimi’ and ‘Jing39’ **(A)**, *ZjHXK* gene family expression levels of ‘Fucuimi’ **(B)** and ‘Jing39’ **(C)** during fruit development, correlation analysis of ‘Fucuimi’ **(D)** and ‘Jing39’ **(E)** between the expression and sugar component. In a, the same lowercase letters indicate no significant difference at the 0.05 level between the two varieties in the same period. YF, E, WM, CR and FR represented fruits at young fruit stage, expanding stage, white mature stage, half-red stage and ripening stage, respectively. The numbers in b & c were FPKM values, the bluer the color, the lower the expression, and the redder the expression. d and e are based on Pearson coefficients, with blue to red indicating negative to positive correlations. *, *p* < 0.05; **, *p* < 0.01.

We determined the sugar composition of cv ‘Fucuimi’ and cv ‘Jing39’ fruit at different developmental stages using gas chromatography. The results showed that the trends in sugar composition changes in cv ‘Fucuimi’ and cv ‘Jing39’ fruit during fruit growth were similarly. However, throughout the entire developmental process, cv ‘Fucuimi’ exhibited significantly higher sugar composition levels compared to cv ‘Jing39’ (**Figure 6A**).

Some *ZjHXK* exhibit similar expression patterns during fruit development of cv ‘Fucuimi’ and cv ‘Jing39’ (**Figures 6B, C**). Among them, the expression level of *ZjHXK3* increases as the fruit grows until maturity stage (WM). In addition, the expression levels of *ZjHXK2*, *ZjHXK4*, *ZjHXK5*, *ZjHXK6*, and *ZjHXK7* decrease as the fruit matures. Notably, *ZjHXK5*, *ZjHXK6*, and *ZjHXK7* consistently exhibit higher expression levels throughout the whole fruit development period. Correlation analysis between sugar content and the expression levels of *ZjHXK* genes in cv ‘Fucuimi’ and cv ‘Jing39’ fruit suggested that *ZjHXK5* and *ZjHXK6* were significantly negatively correlated with total sugar, fructose, glucose, and sucrose content (**Figures 6D, E**).

In addition, we also analyzed the expression levels of *ZjHXK* in six different plant tissues (root, leaf, flower, stem, branch, and fruit) (**Supplementary Figure 6**). We found that *ZjHXK2* was almost not expressed in leaves and fruit but exhibits higher expression in branches. *ZjHXK3* maintains low expression levels throughout the development period. *ZjHXK4* and *ZjHXK5* have higher expression levels in leaves and flowers, while *ZjHXK6* and *ZjHXK7* exhibit higher expression in roots and stems.

### Transient transformation of *ZjHXK5* and *ZjHXK6*

**Figure 7:**
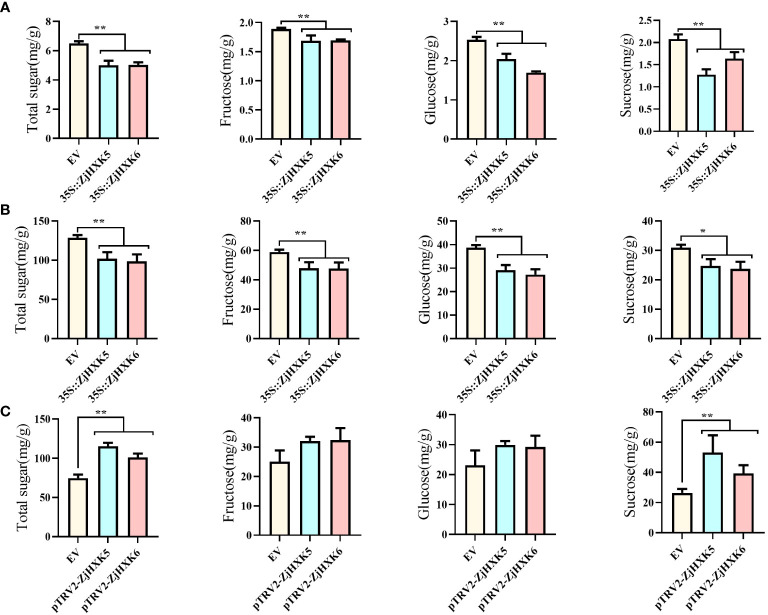
Sugar composition analysis of *ZjHXK5* and *ZjHXK6* in transiently transformed tobacco leaves **(A)** and jujube fruits **(B)**, as well as VIGS-Mediated silencing of jujube fruit gene expression **(C)**. * and ** represent significant differences between treatment and control at 0.05 and 0.01 levels, respectively.

To validate the roles of *ZjHXK5* and *ZjHXK6* in sugar metabolism, we conducted transient transformation in heterologous tobacco leaves and endogenous jujube fruit (**Supplementary Figure 7**). The total sugar, fructose, glucose, and sucrose content in tobacco leaves and jujube fruit were significant decrease after overexpressed *ZjHXK5* and *ZjHXK6* (**Figures 7A, B**). However, through VIGS-mediated silencing of *ZjHXK5* and *ZjHXK6* gene expression, we found an increase in sugar components in jujube fruits, with a significant elevation in both total sugar and sucrose content (**Figure 7C**).

### Stable genetic transformation of *ZjHXK5* and *ZjHXK6*

**Figure 8:**
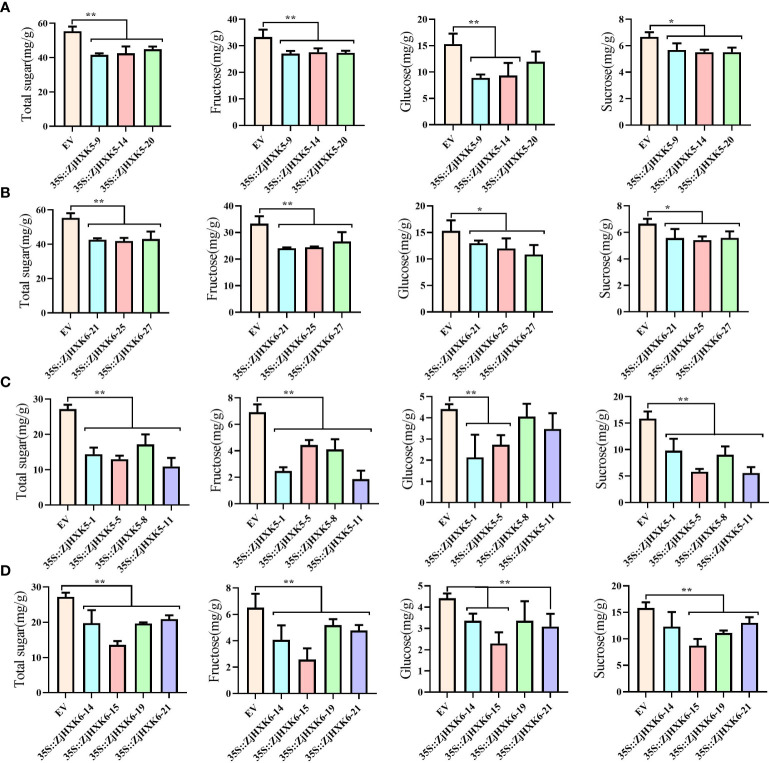
Stable genetic transformation of *35S::ZjHXK5* and *35S::ZjHXK6* in Micro-Tom **(A, B)** and ‘Jing39’ callus **(C, D)**. * and ** represent significant differences between treatment and control at 0.05 and 0.01 levels, respectively.

Through genetic transformation, after stable overexpression of *ZjHXK5* and *ZjHXK6* (**Supplementary Figure 8**), the contents of fructose, glucose and sucrose in tomato fruit and jujube callus were also significantly decreased (**Figures 8A–D**). In addition, we noted that the growth rates of calli decreased, and root length, plant height, leaflet length, and stem thickness of tomato were significantly lower than the control.

### Effect of exogenous MeJA on sugar component content in jujube

**Figure 9:**
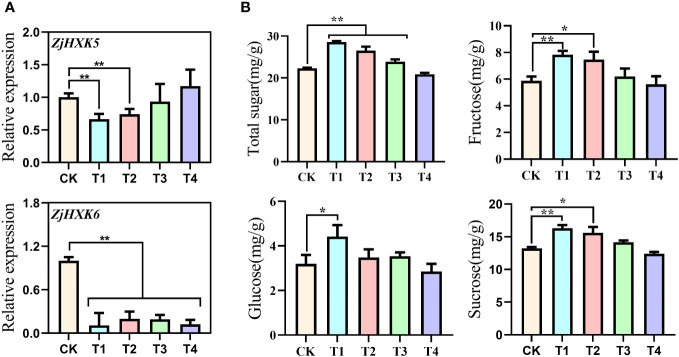
Changes in gene expression levels **(A)** and sugar composition **(B)** of *ZjHXK5* and *ZjHXK6* in ‘Jing39’ callus tissues treated with different concentrations of MeJA. T1, T2, T3, and T4 correspond to MeJA treatments at concentrations of 0.05, 0.10, 0.20, and 0.30 mmol/L, respectively. * and ** represent significant differences between treatment and control at 0.05 and 0.01 levels, respectively.

The promoter regions of *ZjHXK5* and *ZjHXK6* genes contain many cis-regulatory elements responsive to MeJA. Therefore, we designed MeJA treatments with different gradients in jujube calli. The expression of *ZjHXK5* and *ZjHXK6* was significantly down-regulated after treatment with different concentrations of MeJA (**Figure 9A**). Similarly, T1, T2 and T3 treatments significantly increased the total sugar content, and T1 and T2 treatments also had significant effects on the sugar composition (**Figure 9B**). In addition, we observed that as the MeJA treatment concentration increased, the callus browning rate increased, while the growth rate decreased, suggesting that T1 or T2 may the optimal treatment concentrations.

## Discussion

*HXK* gene not only plays a crucial role in various biological processes by catalyzing glucose phosphorylation, but also participates in intracellular signaling, thereby effect plant growth and development (Jang et al., 1997; Granot, 2007). To date, members of the *HXK* gene family have been identified in numerous species, including six in *Arabidopsis* (Karve et al., 2008), ten in rice (Cho et al., 2006), nine in maize (Zhang et al., 2016), seven in cassava, and ten in apple (Zhu et al., 2021). In this study, seven *ZjHXK* genes was identified at gene-wide level. Phosphorylation events are involved in various cellular processes that affect the subcellular localization and stability of target proteins (Johnson, 2009). In this study, a greater number of phosphorylation sites were predicted in ZjHXK proteins, suggesting their susceptibility to post-translational modification events.

The tertiary structure, which involves further twisting and folding on the basis of secondary structure, contributes to a better understanding of gene functionality. Notably, in previous research, the tertiary structure analysis of HXK members was neglected (Huang et al., 2020; Liao et al., 2022). In this study, all ZjHXK proteins possess two distinct tertiary structures with substantial differences between them. This suggests that different HXK tertiary structures may execute distinct functions in various biological processes. This underscores the adaptability and multifunctionality of proteins. It is important to note that the existence of these two tertiary structures may potentially lead to divergent enzymatic functions.

Previous research has shown that the characteristics of gene intron/exon sequences are crucial for understanding gene function and evolutionary relationships (Liao et al., 2020). In this study, it was found that the number of introns and coding sequences (CDS) in *ZjHXK* gene family members was consistent, further indicating the high conservation in the evolution of *ZjHXK* genes. The results of conserved motif analysis support this result, as many HXK family members share the same conserved motifs. This is one of the main reasons why some genes cluster together in phylogenetic trees. According to the phylogenetic analysis, HXK is classified into subfamilies A, B1, B2, and C2, corresponding to subfamilies V, IV, I, and II in *Arabidopsis*, which was consistent with previous studies (Dai et al., 2002; Kim et al., 2013; Geng et al., 2017). The jujube genome has undergone frequent interchromosomal fusions and segmental duplications, but a recent whole-genome duplication has not occurred (Liu et al., 2014). Multiple collinearity analysis of HXK suggested that HXK had originated through duplication events among its own family members. Intraspecific genome collinearity analysis of HXK revealed two pairs of collinear gene pairs, ZjHXK1 and ZjHXK5, and ZjHXK6 and ZjHXK7, indicating homologous genes on different chromosomes during the course of evolution. These genes further confirmed the high conservation of the HXK gene family. Inter-specific genome collinearity showed that four ZjHXK gene family members exhibited collinearity with three *Arabidopsis* HXK genes, suggesting some similarity between jujube and *Arabidopsis* HXK genes. It is also possible that duplicate genes may have been altered or lost during the evolutionary process in different species (Yang et al., 2023).

Promoter cis-regulatory element analysis revealed that all members of the *ZjHXK* family possess multiple hormone-responsive elements, light-responsive elements, and stress-responsive elements. However, there were significant differences in the types and number of cis-regulatory elements in the same orientation among these family members. These findings suggest that multiple homologous genes have gradually evolved during the process of plant development, thereby avoiding situations in which mutations in a single gene result in the loss of function and slow down or halt growth (Magadum et al., 2013). In addition, *ZjHXK* genes were also involved in plant growth and development. Further analysis of cis-elements highly related to sugar components indicated that *ZjHXK* promoters were predominantly associated with hormone-regulatory elements, with ABRE elements being the most common. Overexpression of *AtHXK* in *Arabidopsis* led to increased sensitivity to cytokinin in cell division, implying that *ZjHXK1* in jujube may regulate plant hormone sensitivity.

As is widely recognized, hexokinase (HXK) is known to participate in the regulation of fruit sugar content by catalyzing the phosphorylation of hexose, thereby influencing sugar metabolism and accumulation. Its activity and expression levels are likely to play a pivotal role in determining fruit sugar content and sweetness. Previous studies have indicated that jujube is a fruit with relatively high sugar content (Bartolozzi et al., 1997; Sparkes et al., 2006; Sun et al., 2015). By assessing the gene expression levels and sugar component changes in high and low sugar jujube fruits at various developmental stages, we can gain a more comprehensive understanding of the mechanisms underlying sugar accumulation. Gene expression profile analysis revealed that the majority of *ZjHXK* genes exhibit a downregulation in expression levels as the fruit matures, while the trend in sugar component content is opposite to this. Correlation analysis suggests that *ZjHXK5* and *ZjHXK6* genes may play a significant role in the high-sugar accumulation phenotype of jujube, consistent with functional studies in pear (Zhao et al., 2019) and apple (Zhu et al., 2021). One of the most interesting results is that *ZjHXK* genes were expressed in multiple tissues, but their expression levels vary significantly, indicating that the *ZjHXK* genes perform distinct functions in different organs.

Due to the difficulty of stable genetic transformation in jujube, even if transgenic plants are obtained, it will take 3-5 years to fruit. Therefore, most studies of gene function rely on instantaneous transformation of callus or stable expression in model plants. Some fruit trees are also identified gene function by this way (Anjanappa and Gruissem, 2021). In this study, *ZjHXK5* and *ZjHXK6* were been confirmed that can negatively regulate sugar metabolism, consistent with previous studies, such as transgenic tomato leaves expressing *AtHXK1* exhibited a decrease in photosynthetic efficiency, accelerated senescence, and varying degrees of reduction in young fruit weight, starch content, and soluble sugar content in mature fruits (Dai et al., 1999). It can be inferred that *ZjHXK5* and *ZjHXK6* accelerate the phosphorylation of hexoses, resulting in reduction of sugar composition, and thus regulate plant growth and development. In addition, a large number of studies have shown that the expression of HXK genes in plants is influenced by certain exogenous hormones (Hitoshi, 2006; Pimenta Lange and Lange, 2006; Mishra et al., 2009). Both the *ZjHXK5* and *ZjHXK6* promoters contain MeJA-responsive elements, including TGACG-motif and CGTCA-motif. Upon subjecting jujube callus tissues to MeJA treatment, it was observed that the expression of *ZjHXK5* and *ZjHXK6* genes was significantly downregulated. Simultaneously, sugar components exhibited a significant increase under MeJA treatment at concentrations of 0.05 mmol/L and 0.10 mmol/L. This suggests that *ZjHXK5* and *ZjHXK6* were downregulated under MeJA induction and can effectively regulate sugar accumulation at suitable concentrations. MeJA treatment before fruit picking can not only increase the soluble solids and total sugar content of the fruit (Li et al., 2023), but MeJA treatment after fruit picking can also increase the antioxidant capacity and phenolic content of the fruit, thereby extending the shelf life of the fruit (Wang et al., 2021). Although the present study does not provide a detailed explanation of the underlying mechanisms, it holds valuable reference value for a deeper understanding of the molecular mechanisms governing sugar composition regulation in jujube fruits. Furthermore, it offers a new avenue of investigation into the *HXK* gene family.

## Conclusions

In this study, we have provided a comprehensive understanding of the identification of the *HXK* gene family in jujube, as well as the identified of key genes related sugar metabolism. Seven *ZjHXK* genes were identified in the jujube genome. All *ZjHXK* genes exhibit similar gene structures, conserved motifs, and subcellular localization. Many common and unique cis-elements were identified within the *ZjHXK* promoter regions. Additionally, a phylogenetic tree of HXK genes was constructed, classifying them into five subgroups. Through the correlation analysis of *ZjHXK* expression levels and sugar component content, we found that *ZjHXK5* and *ZjHXK 6* were the key genes regulating sugar. The sugar content was decreased after overexpression in jujube calli, tobacco leaf and tomato fruit. Transient silencing of *ZjHXK5* and *ZjHXK6* genes results in a significant increase in sucrose and total sugar content. Furthermore, *ZjHXK5* and *ZjHXK6* were downregulated under MeJA treatment, which significantly enhanced sugar component content. In summary, our study lays the foundation for further exploration of the molecular mechanisms of sugar metabolism in jujube.

## Data availability statement

The original contributions presented in the study are included in the article/**Supplementary Material**. Further inquiries can be directed to the corresponding authors.

## Author contributions

PT: Writing – original draft. GL: Writing – review & editing. DL: Resources, Writing – original draft. XZ: Visualization, Writing – original draft. WZ: Data curation, Writing – original draft. QX: Writing – review & editing. CW: Supervision, Writing – review & editing. JW: Writing – review & editing.
