# Supplementary material for: ZjHXK5 and ZjHXK6 negatively regulate the sugar metabolism of Ziziphus jujuba Mill
Source: Front Plant Sci. 2024 Feb 12;15:1335120. doi: 10.3389/fpls.2024.1335120 (PMC10895003; doi:10.3389/fpls.2024.1335120)
Supplement: Supplementary file 1 [file DataSheet_1.doc]

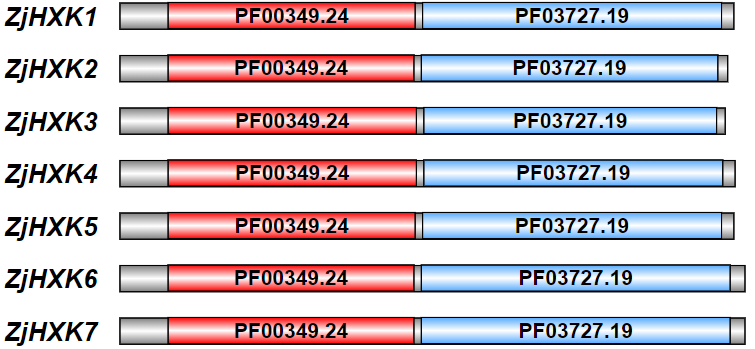


Supplementary Fig. 1 Protein conserved domain of HXK gene family in Jujube. Different colored boxes represent different protein sequences, gray boxes represent the full length of the protein, red boxes represent the hexokinase-1 domain (ID: PF00349.24), and blue boxes represent the hexokinase-2 domain (ID: PF03727.19).


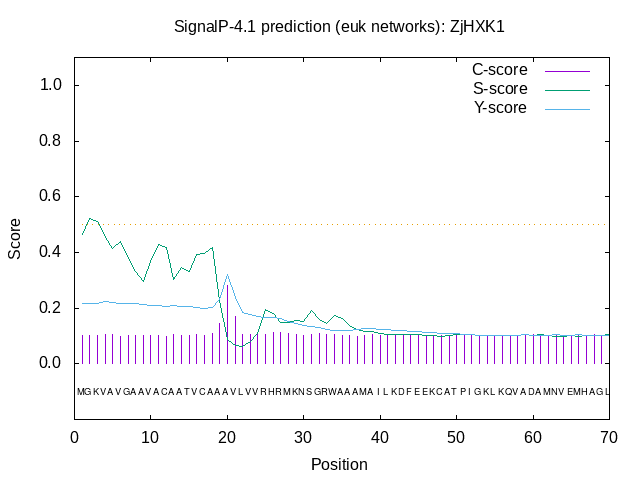

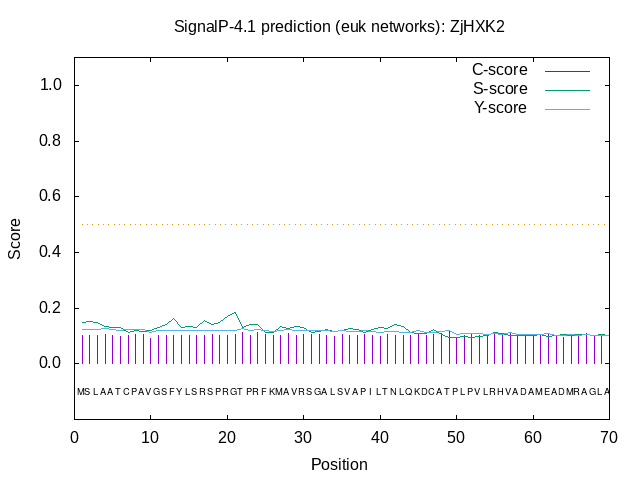

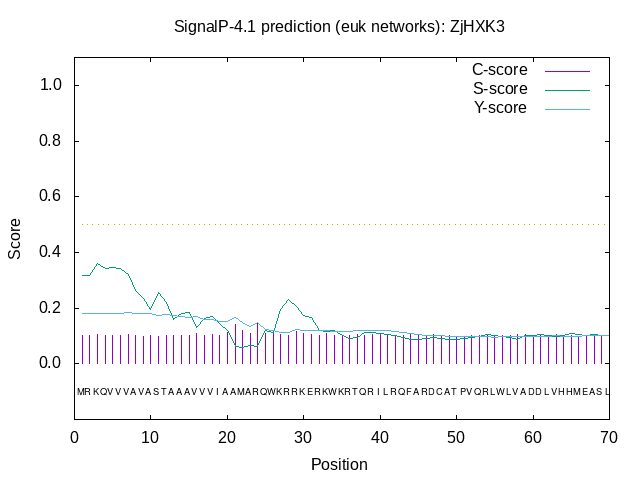

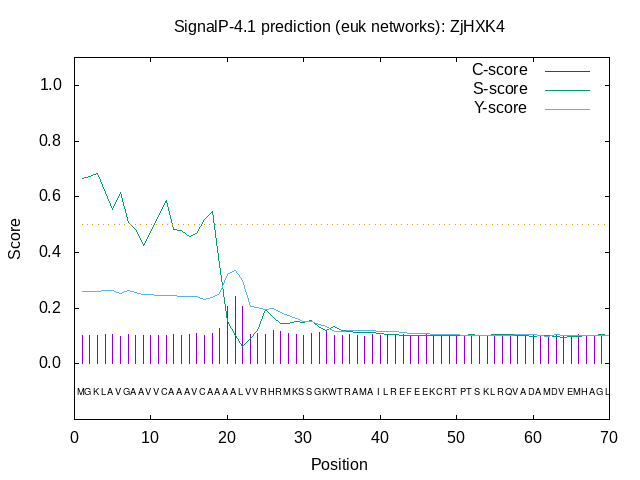

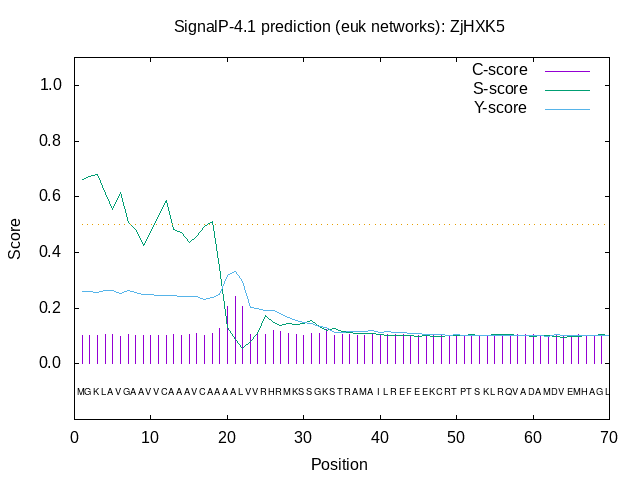

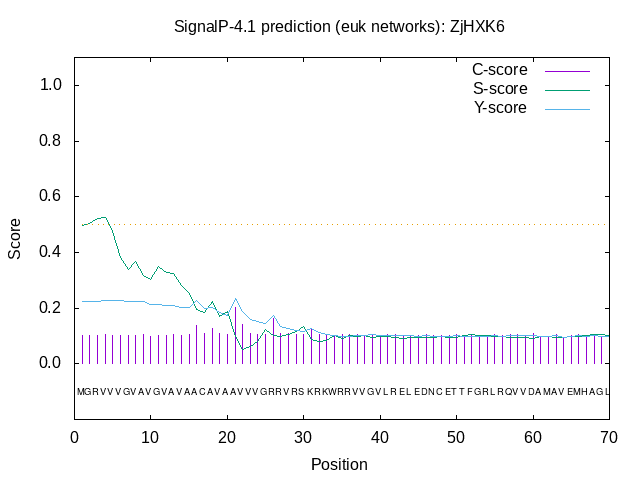

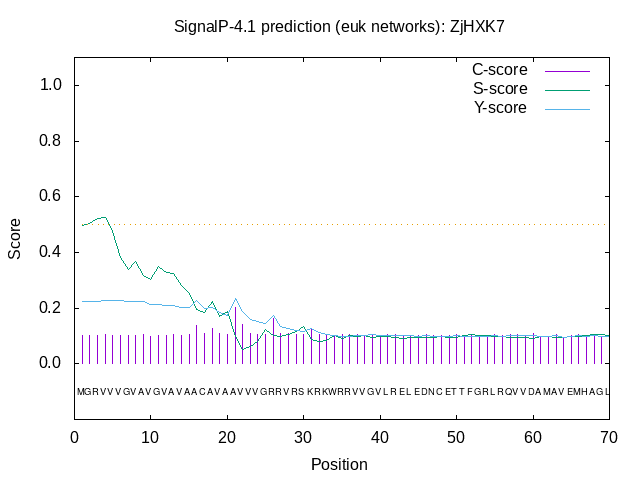


Supplementary Fig. 2 Analysis of signal peptide of HXK gene family in red jujube.


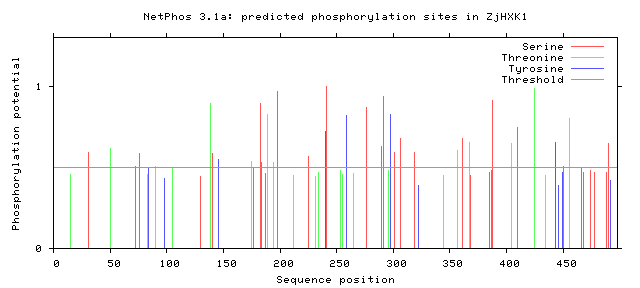

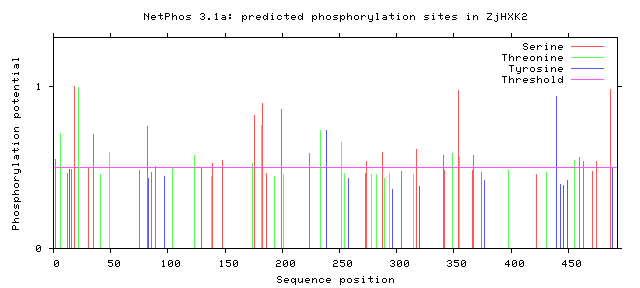

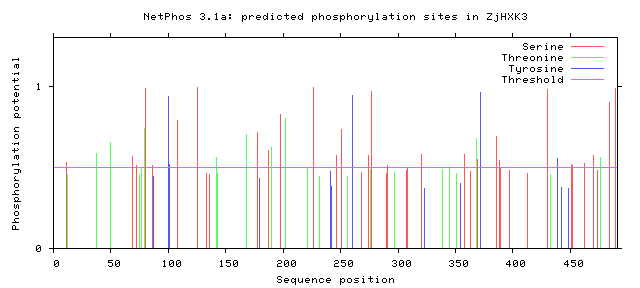

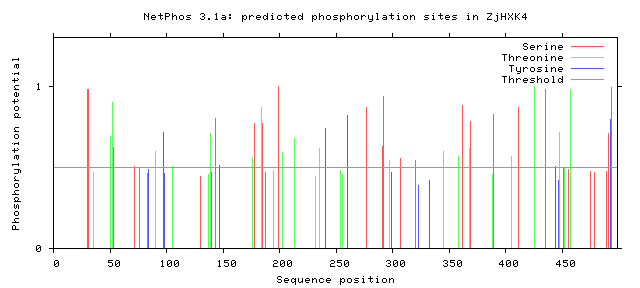

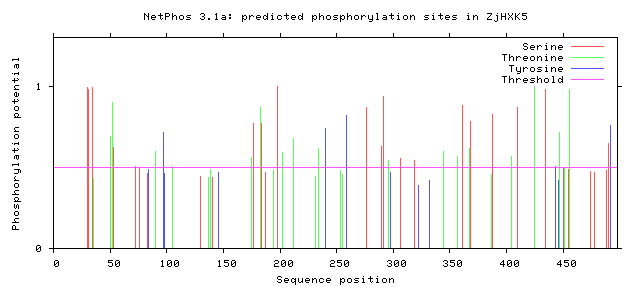

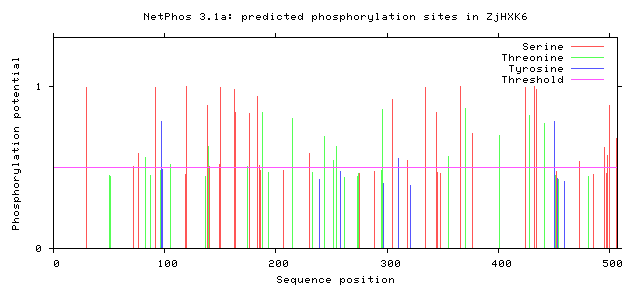

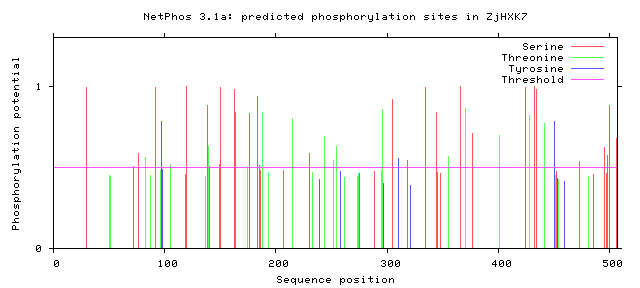


Supplementary Fig. 3 Analysis of phosphorylation sites of HXK gene family in red jujube.


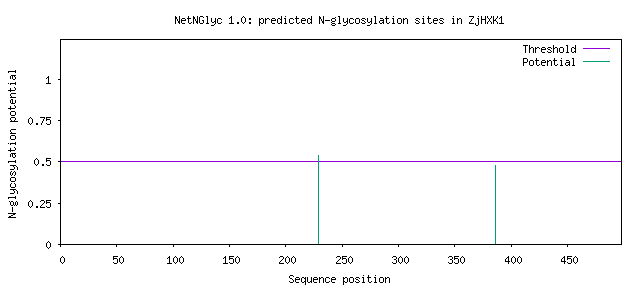

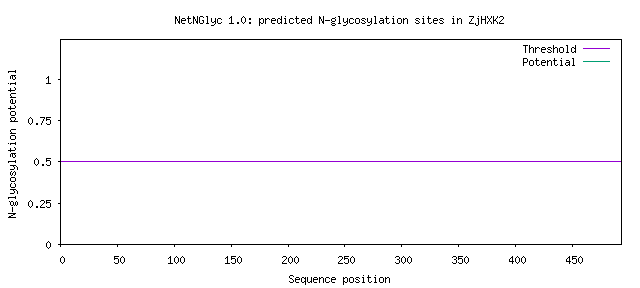

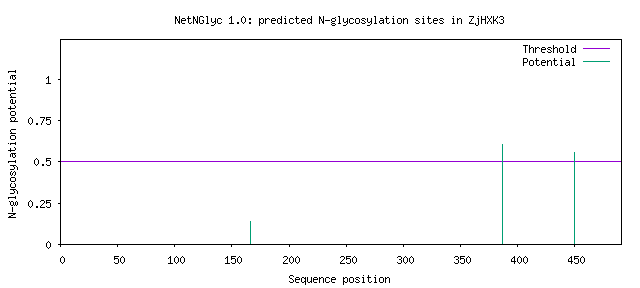

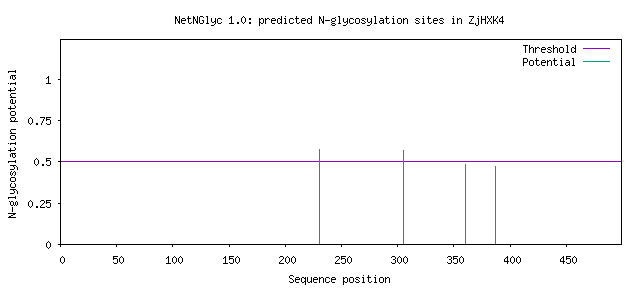

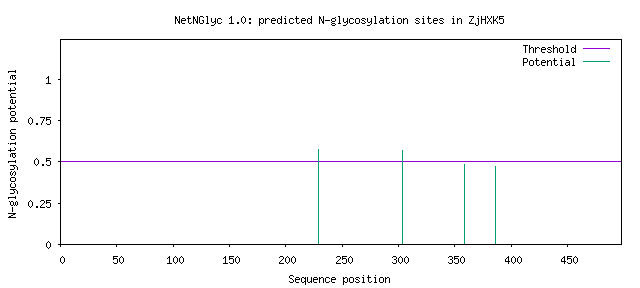

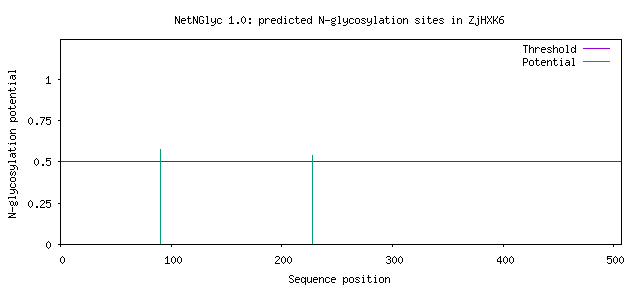

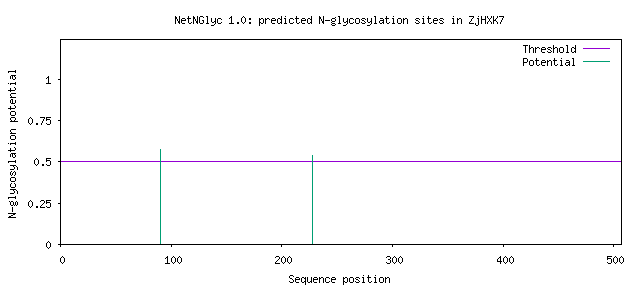


Supplementary Fig. 4 Analysis of glycosylation sites of HXK gene family in jujube.


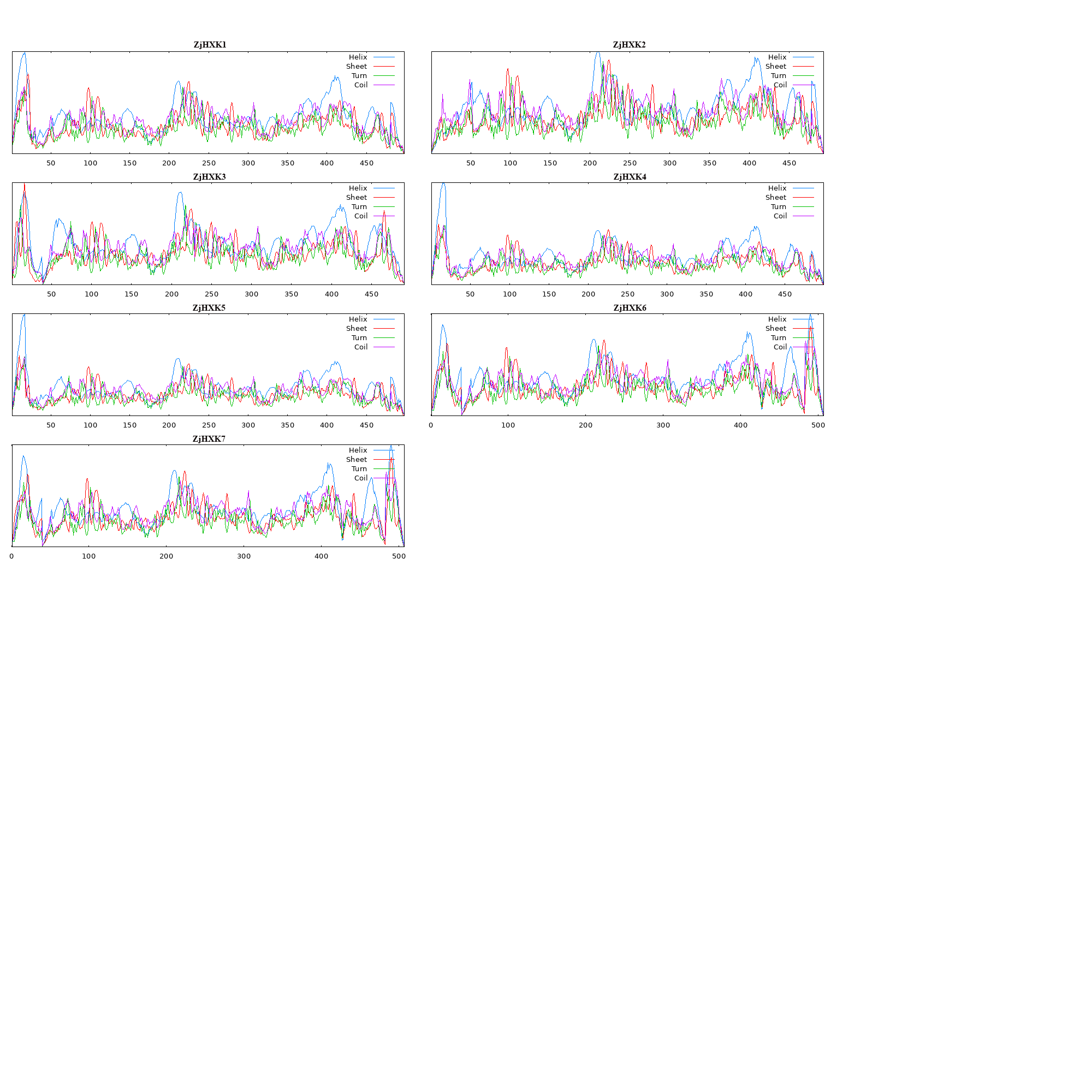


Supplementary Fig. 5 Analysis of secondary structure of HXK gene family in jujube.


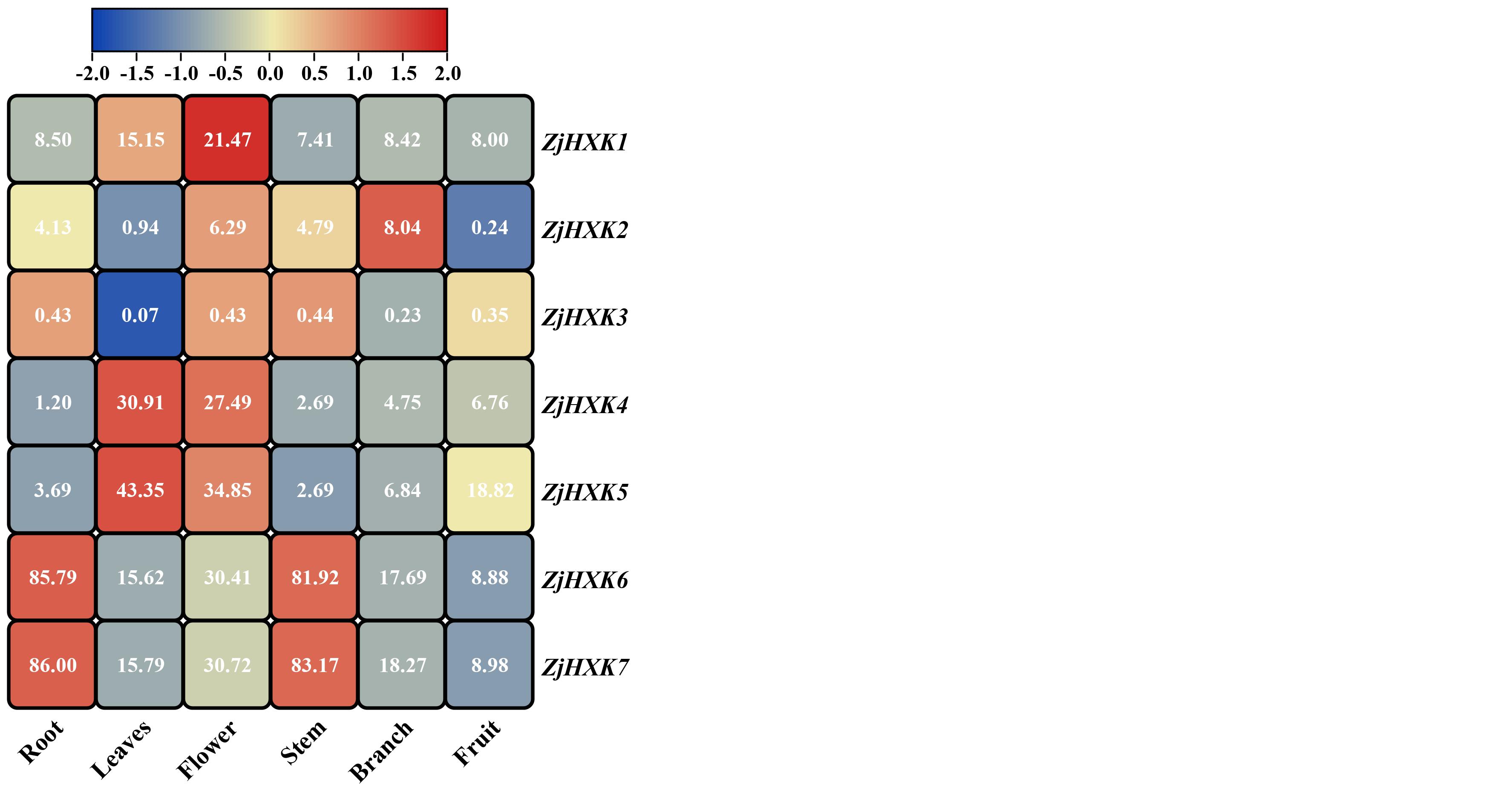


Supplementary Fig. 6 Expression patterns of *ZjHXK* gene in different tissues.


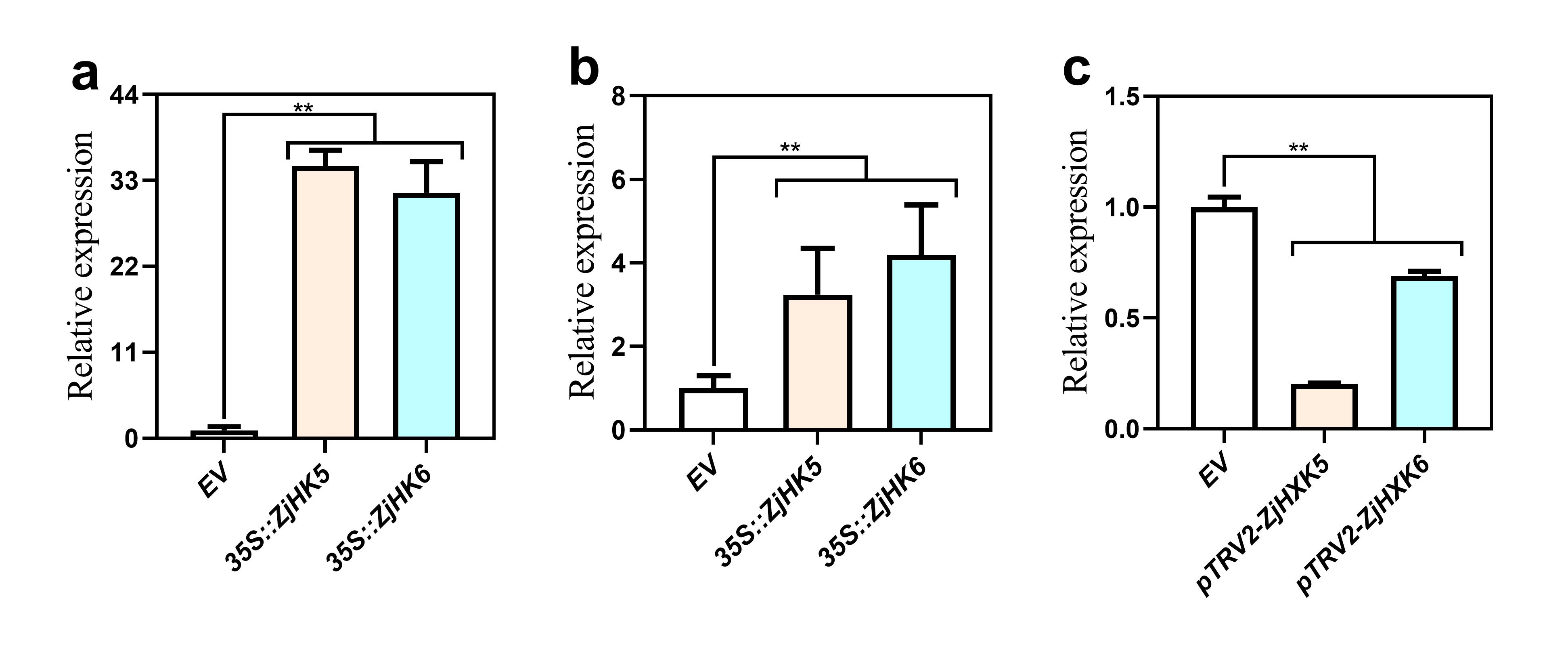


Supplementary Fig. 7 qPCR Validation of transient transformation of *ZjHXK5* and *ZjHXK6* in tobacco leaves (a) and jujube fruits (b), as well as VIGS-Mediated silencing of jujube fruit gene expression (c).


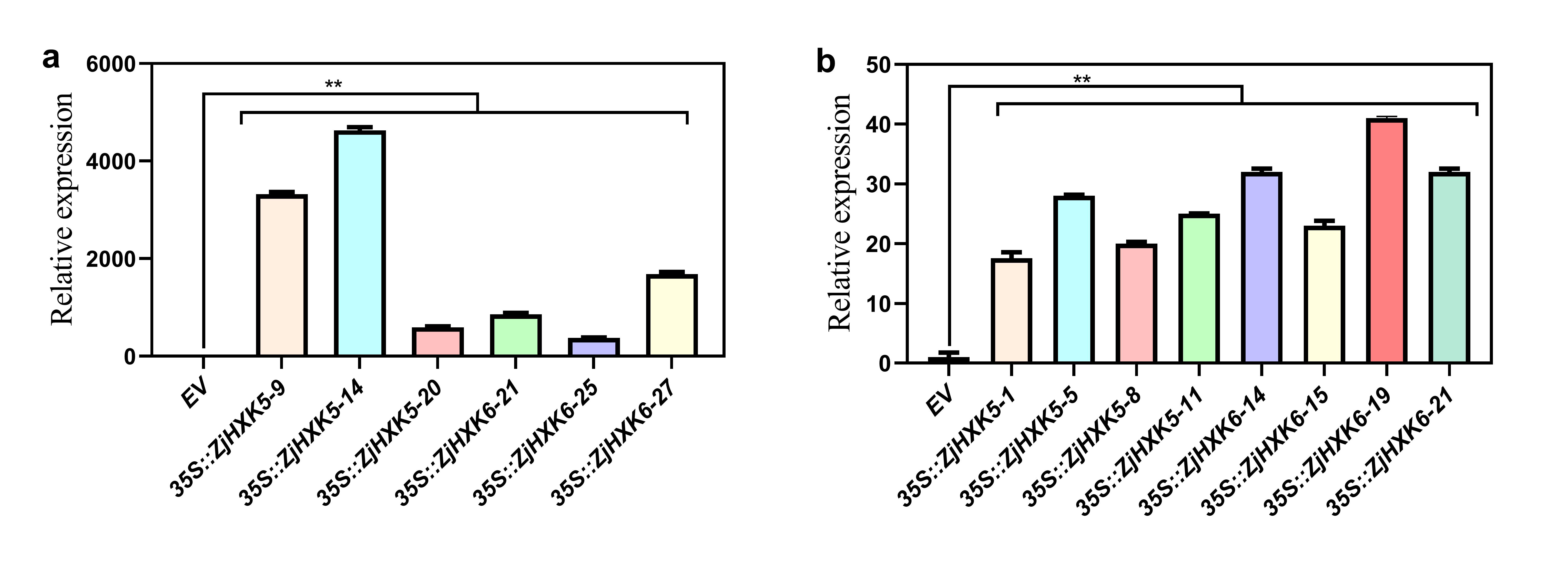


Supplementary Fig. 8 qPCR validation of stable genetic transformation of *35S::ZjHXK5* and *35S::ZjHXK6* in tomato (a) and jujube callus (b).
